# Prevalence and Presentation of Lower Limb Neurovascular Complications in Children With Diabetes: A Systematic Review With Proportion Meta-Analysis

**DOI:** 10.1155/pedi/7664860

**Published:** 2025-06-23

**Authors:** Iris Syifaa Fathiah Irwandy, Fiona Hawke, Andrea Coda, Richard G. McGee, Stewart Birt, Antoni Fellas

**Affiliations:** ^1^School of Health Sciences, College of Health, Medicine and Wellbeing, The University of Newcastle, Newcastle, NSW, Australia; ^2^Equity in Health and Wellbeing Research Program, Hunter Medical Research Institute (HMRI), Newcastle, NSW, Australia; ^3^Department of Paediatrics, Campbelltown Hospital, Campbelltown, Sydney, NSW, Australia; ^4^School of Medicine, Western Sydney University, Campbelltown, Sydney, NSW, Australia; ^5^School of Medicine and Public Health, College of Health, Medicine and Wellbeing, The University of Newcastle, Newcastle, NSW, Australia; ^6^Department of Paediatrics, Gosford Hospital, Gosford, NSW, Australia

## Abstract

**Introduction:** Disorders of the lower limb are common in people with diabetes and may result in significant long-term complications. Currently, most evidence for lower limb complications in children with diabetes relies on infrequent clinical observations and occasionally invasive assessments that can cause discomfort. Clinical guidelines do not provide explicit guidance on frequency of neurovascular assessments for children with diabetes.

**Aim:** To systematically review the prevalence and presentation of clinical neuropathy and vasculopathy in children with diabetes.

**Methods:** A systematic search of the literature was conducted in March 2024 using the EMBASE, Cochrane, PubMed (including MEDLINE) and CINAHL databases. At least two authors independently screened studies for inclusion and assessed methodological quality for each paper using the downs and black quality appraisal checklist. Extracted data was summarised and tabulated. Meta-analysis was also performed.

**Results:** Eighteen studies with 3533 participants were included. Participants were aged 3.5–18 years, and 95% had type 1 diabetes, while 5% had type 2 diabetes. These studies used a range of assessments, including monofilament, vibrioception, sharp-blunt discrimination, temperature perception, reflexes, muscle strength and tone, joint perception, pedal pulses, and ankle brachial index (ABI). Prevalence of clinical neuropathy ranged from 0% to 57.1% with a prospective cohort study reporting a 2.6-fold increase in 5 years, while prevalence of vascular complications ranged from 0% to 37.8%. The meta-analysis reported 0%–7% of children could report abnormality on assessments for neuropathy.

**Conclusion:** The broad range may be due to heterogeneous methodological designs and diagnostic assessments, and potentially inadequate reporting. There is emerging evidence that children with diabetes may develop complications before age 18, which provides impetus for clinical caution. Higher quality evidence, particularly from longitudinal studies, is required to guide clinical screening for lower limb vascular and neurological complications in this vulnerable and growing paediatric population.

## 1. Introduction

Disorders of the lower limb are common in people with diabetes and can result in significant long-term complications [1, 2]. Diabetic neuropathy is defined as a neurodegenerative disorder affecting the somatic and/or autonomic peripheral nervous system, in the absence of other secondary causes [3]. Up to half of individuals with type 1 and type 2 diabetes will develop diabetic neuropathy, with disease duration and suboptimal glycaemic control being recognised risk factors [4]. Interestingly, nerve damage may occur prior to individuals being diagnosed with diabetes, with evidence suggesting a link between prediabetes and early small-fibre symptoms [4]. It is unclear if similar findings are present in children.

Micro (typically seen in diabetes as damage to small blood vessels in the eyes, kidneys and nerves) and macrovascular complications (affecting large blood vessels, which may lead to coronary artery disease, peripheral arterial disease and cerebrovascular disease) are also well recognised with diabetes [5]. Evidence suggests that children with type 1 diabetes may have a higher prevalence of peripheral arterial disease than age-matched controls [6]. While 60.1% of children diagnosed with type 2 diabetes have been identified as having at least one microvascular complication by young adulthood, highlighting the importance of regular and adequate screening [7]. Long-term complications of diabetes are associated with disability, reduced quality of life, healthcare costs and significant rates of morbidity and mortality [8, 9]. In less-resourced countries, the financial burden of managing diabetes is even more challenging, with minimal care for a child with type 1 diabetes costing up to 56% of an average annual income, making additional complications prohibitively expensive [10].

Despite the high prevalence of these complications, current screening methods remain inadequate in early detection and management. Most of the evidence for the presence of lower limb complications in children with diabetes is based on nerve conduction studies (NCS), which are the gold standard in detecting disturbances in nerve health in people with diabetes. However, they are rarely utilised clinically, particularly in a paediatric setting, as they cause pain or discomfort often not tolerated by children [11, 12]. Paediatric endocrinology teams will therefore opt for painless and more accessible clinical assessments, such as those conducted in podiatry clinics, to screen for lower limb complications.

Neurovascular screening of the lower limb is a collection of non-invasive clinical assessments used to evaluate the health of the neurological and vascular systems. Assessments can include, but are not limited to, using Doppler ultrasound and toe/ankle pressure technology to assess peripheral arterial flow to the feet and toes, as well as screening for neurological deficit with monofilament, pain and vibration perception tests. According to the International Working Group on the Diabetic Foot (IWGDF) clinical guidelines, it is recommended for an adult diagnosed with diabetes to obtain annual neurovascular screening for identification of an at-risk of foot ulceration [13, 14]. This is also consistent with the recently published national guidelines recommended by Diabetes Australia [15]. However, these recommendations do not provide explicit information on frequency of neurovascular assessments for children with diabetes. The 2022 ISPAD guidelines note a lack of available symptom scores to assess diabetic neuropathy in children. However, recommended screening for peripheral neuropathy in children with type 1 diabetes from puberty or from ages 11 years with 2 to 5 years of diabetes duration to be repeated annually using temperature or pinprick sensation, vibration and ankle reflexes. They also recommend screening for diabetic neuropathy in type 2 diabetes at diagnosis and be repeated annually. The ISPAD guidelines also suggested screening for macrovascular complications using blood pressure and lipid profiles, but that routine screening for other markers of macrovascular complications outside the research setting is unclear [16]. Therefore, the research question developed for this systematic review is: what is the prevalence and presentation of clinical neuropathy and vasculopathy in children with type 1 and type 2 diabetes? By evaluating the available evidence, we seek to provide further clarity on the occurrence and characteristics of these complications in the paediatric population, to better inform clinical practice guidelines in children with all forms of diabetes mellitus.

## 2. Methods

### 2.1. Studies and Participants

This review included studies reporting the prevalence of lower limb neuropathy and vasculopathy in children and adolescents up to the age of 18 years, diagnosed with type 1 or type 2 diabetes and was utilising podiatric clinical methods of diagnosing neuropathy and vasculopathy, that is, monofilament, tuning fork, neurothesiometer, biothesiometer, patella and Achilles reflex, sharp-blunt differentiation, pulse palpation, toe brachial index, ankle brachial index (ABI) and Doppler waveform and sound analysis. Studies, which included patients with other dermatological and musculoskeletal-related complications, any additional rheumatological and/or other medical conditions associated with lower limb neurovascular complications were excluded. Furthermore, studies diagnosing subclinical neuropathy via only NCS were excluded. All types of observational studies were considered for inclusion. Poster abstracts and conference proceedings were excluded. There were no restrictions on setting, for example, inpatient or outpatient. Non-English language articles were considered for inclusion. Abstracts that were non-English were screened using a university library translation service; however, this was never required.

### 2.2. Search Strategy

The following databases were searched on 15th March 2024: MEDLINE (January 1966–15th March 2024), EMBASE (January 1980–15th March 2024), Cochrane Central Register of Controlled Trials (CENTRAL) (The Cochrane Library, latest issue), PubMed (which includes MEDLINE) (January 1966–15th March 2024) and CINAHL (1982–15th March 2024).

The following are the combined keywords used to perform the literature search: (“diabetes” OR “diabetes type 1” OR “diabetes type 2” OR “diabetes mellitus” OR “juvenile diabetes”) AND (“child” OR “child, preschool” OR “adolescent”) AND (“lower limb” OR “lower extremity” OR “ankle” OR “foot” OR “feet” OR “diabetic foot” OR “neuropathy” OR “diabetic neuropathy” OR “vasculopathy” OR “peripheral arterial disease” OR “peripheral vascular disease” OR “diabetic foot ulcer”). The search syntax is presented in Table S1. All papers were then imported into covidence (https://www.covidence.org) and duplicates were excluded.

Two authors (Iris Syifaa Fathiah Irwandy and Antoni Fellas) independently screened the titles and abstracts of all articles identified in the search. Both authors then independently screened full-text versions of potentially eligible studies. Authorship and results were not masked. If a disagreement arose between the two authors regarding the full-text inclusion, a third reviewer (Andrea Coda) acted as an arbitrator. If the disagreement could not be resolved successfully by the arbitrator, the study author was to be contacted to settle the disagreement; this process was not required. If a disagreement was not successfully resolved, the disagreement was to be acknowledged and stated in the review; again, this was not required. Standardised pilot-tested forms were used when extracting the data from included full-text articles.

The following data were extracted: author, year of publication, year of data collection, country, recruitment methods, description of participants and results. One author (Iris Syifaa Fathiah Irwandy) extracted relevant data, and a second author (Antoni Fellas) checked all the extracted data. Any disagreements were discussed between authors (Iris Syifaa Fathiah Irwandy and Antoni Fellas), and if required, discrepancies were arbitrated by another review author (Fiona Hawke), though this was not required.

This review included studies with enough data for analysis either from a published report or provided by the author. If there was any absent or uncertain data, the study author was contacted via email by one review author (Iris Syifaa Fathiah Irwandy). If there was no reply within 2 weeks, another follow-up email was sent. If no response was received to the initial follow-up email from the study author, then the study was excluded from the analysis. These exclusions were reported in the table of excluded studies (Table S2) in supporting information. If the author replied to the email, then a timeframe of 3 weeks was permitted for data retrieval.

### 2.3. Quality Appraisal

The Downs and Black [17] quality appraisal checklist was used for quality assessment. Each study was appraised by at least two authors independently (Iris Syifaa Fathiah Irwandy, Antoni Fellas, Fiona Hawke, Andrea Coda, and Richard G McGee). Any discrepancies were discussed between authors, and if arbitration was required, a third review author (Antoni Fellas) made the final decision.

### 2.4. Statistical Analysis

A proportion meta-analysis was conducted using STATA 18. Studies that investigated the same outcome, such as vibration perception, were pooled. The number of participants and abnormal events were extracted from each study and analysed using the Freeman–Tukey double-arcsine-transformed proportion. This analysis is particularly suitable for proportions that are expected to be close to 0 or 1. Forest plots were then generated, and the meta-analysis results presented an overall (mean) proportion and confidence intervals.

## 3. Results

**Figure 1:**
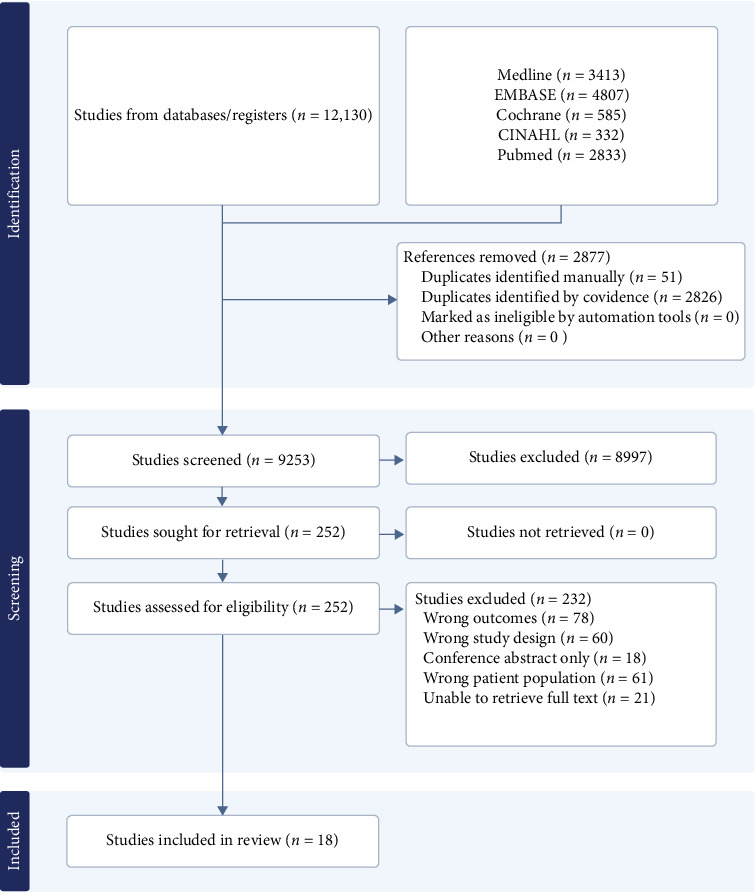
PRISMA flow diagram.

Figure 1 depicts the PRISMA flow diagram. A total of 12,130 papers were located and imported into covidence. In total, 2877 duplicates were excluded. A further 8997 articles were removed based on their titles and abstracts. The full text of 232 articles was reviewed for final inclusion, and 212 of these were excluded (see Figure 1). Finally, a further two studies (Almenabbawy et al. 2019 and Demirel et al. 2013) were excluded as the authors were unable to provide information on what clinical assessments they conducted or provide their respective results after several attempts to contact the authors. Only Nelson et al. [18] and Chuback et al. [19] replied to our queries. Overall, 3602 participants with type 1 or 2 diabetes across 18 studies were included in this review, with an age range of 3.5–18 years old [5, 6, 11, 18–32]. Fifteen out of the 18 studies used a cross-sectional study design, with seven having controls, two used a descriptive retrospective design, and one was a prospective cohort study [5, 6, 11, 18, 19, 21, 23–31]. Most studies recruited participants from a single outpatient paediatric endocrinology department, and two were multicentre. Participants were recruited from Taiwan, Australia, Austria, Canada, Finland, Germany, India, Iran, Istanbul, Nigeria, Sweden, and the United Kingdom.  Table 1 details the characteristics of the included studies.

**Figure 2:**
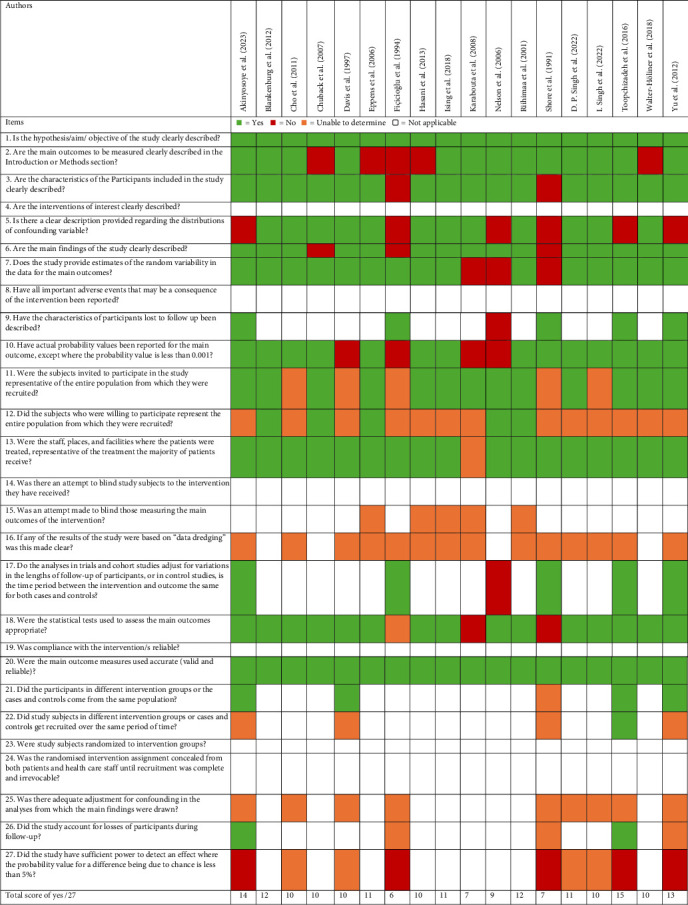
Quality appraisal summary.

This review had used “not applicable” (blank) for the mention of interventions in the risk of bias assessment, as measuring prevalence does not change the outcome of the participants. This includes the use of questions 4, 8, 14, 19, 23 and 24. This was with an exception of questions 15, 17, 21 and 22. This review could not identify any of the studies' attempts to blind those measuring the outcomes, whether any results were based on data dredging and whether there was adequate adjustment for confounding in the analyses from which the main findings were drawn. All studies were able to clearly describe the objective of the study and used accurate main outcome measures. Most studies clearly described the main outcomes to be measured in the introduction or methods section, the characteristics of the participants included in the study, the main findings and the characteristics of participants lost to follow-up were described. Majority of the studies also provided a clear description provided regarding the distributions of confounding variable, estimates of the random variability in the data for the main outcomes, had actual probability values been reported for the main outcome (except where the probability value is <0.001) and used appropriate statistical tests used to assess the main outcomes. Most studies had invited subjects to participate in the study being the representative of the entire population from which they were recruited and the staff, places, and facilities where the patients were treated was representative of the treatment the majority of patients had received, however, this review with more than half of the studies were unable to determine if subjects who were willing to participate represented the entire population from which they were recruited. Results of risk of bias assessment are presented in Figure 2.

### 3.1. Neuropathy

Sixteen studies described six clinical assessments to detect neuropathy in children with diabetes, including the touch perception test, vibration perception test, pain perception test, thermal perception test, muscle strength test, muscle tone, deep tendon reflex and hallux joint position perception [11, 18–32].

#### 3.1.1. Touch Perception Test (TPT)

The monofilament or the touch perception test was carried out in 11 studies [11, 18, 19, 24–27, 29–32].

Blankenburg et al. [11] conducted the TPT assessment on a cohort of 45 participants with mean age of 13.2 years, mean type 1 diabetes duration of 6.7 years and mean HbA1C of 8.28%, compared to 45 aged and gender matched controls showed that participants without diabetes exhibited 3 times higher tactile detection thresholds. This is supported by Karabouta et al. [26], Nelson et al. [18], Hasani et al. [24] and Walter-Höliner et al. [32] with a total cohort of 254 participants, which noted prevalence of abnormal results ranging from 7.9% to 57.1%. These studies had a median duration of type 2 diabetes of 1.8 years or range mean of type 1 diabetes duration of 3.8–8.1 years, range of mean age of 11.9–13.7 years and range of mean HbA1C of 8.1–9.0%. Contrasting results were highlighted by Chuback et al. [19], Ising et al. [25], Riihima et al. [27], Singh et al. [29], Singh et al. [30] and Toopchizadeh et al. [31] as their respective studies recorded no abnormalities.

None of the studies reported whether participant characteristics were associated with higher rates of abnormal touch perception.

**Figure 3:**
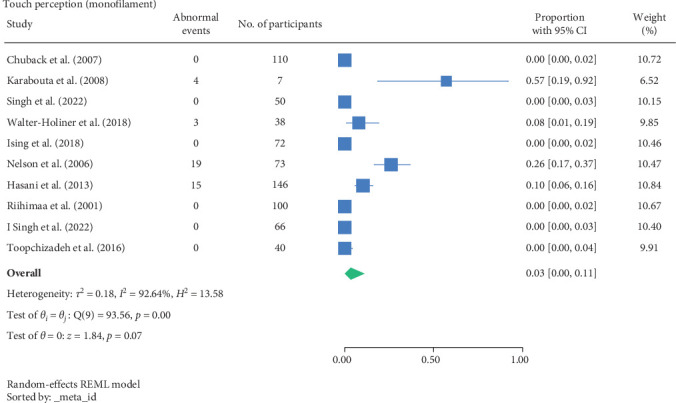
Meta-analysis of abnormal touch perception events.

The result of the proportion meta-analysis of the 10 studies evaluating abnormal touch perception events can be seen in Figure 3. The overall (mean) proportion of participants exhibiting an abnormal touch perception test result was 0.03 with a CI of [0.00, 0.11], indicating that 3% of children with type 1 or 2 diabetes mellitus could exhibit an abnormal touch perception.

#### 3.1.2. Vibration Perception Test

Vibration perception test was carried out in 16 studies [11, 18–32]. A variety of tools were used in this assessment, such as the tuning fork, biothesiometer, a vibrosense metre device and a Vibrometry system 9589. All studies which used a tuning fork detected no abnormalities or no statistically significant difference between cases and controls [11, 19, 23, 26, 29]. Four studies employed the biothesiometer [20–22, 28].

Davis et al. [21] measured vibration perception threshold (VPT), which noted 9.1% of 307 participants (with mean age 13.3 years, mean type 1 diabetes duration of 5.1 years and mean HbA1C 10.7%) had abnormal results. Among these 9.1% participants, they had a mean age of 13.4 years, mean type 1 diabetes duration of 5.1 years and mean HbA1C 11.2%, where 11 were younger than 11 years old and eight were prepubertal. This contrasts with Shore et al. [28], where no abnormalities were recorded among the 50 participants with mean age of 13.1 years and median duration of type 1 diabetes of 49 months.

Ising et al. [25] opted for the VibroSense Metre device at the plantar aspect of the first and fifth metatarsal heads of the right foot where 18% had at least one pathological site on the foot for VPT, 6% had at least 2 sites and 4% had at least 3 sites among 72 participants with median age, type 1 diabetes duration and age at disease onset being 12.8, 5.3 and 6.9 years, respectively.

Blankenburg et al. [11] reported that abnormal VPTs occurred exclusively in patients with longer diabetes duration, whilst Ising et al. [25] reported no statistical differences. Furthermore, Ising et al. [25] report no statistical difference in gender, HbA1c, height, weight, and BMI; however, noting that participants presenting with at least one pathological site compared to subjects with no pathological sites had a more advanced age of disease onset. Riihima et al. [27] reported a slight difference in VPT only after puberty in patients and controls.

**Figure 4:**
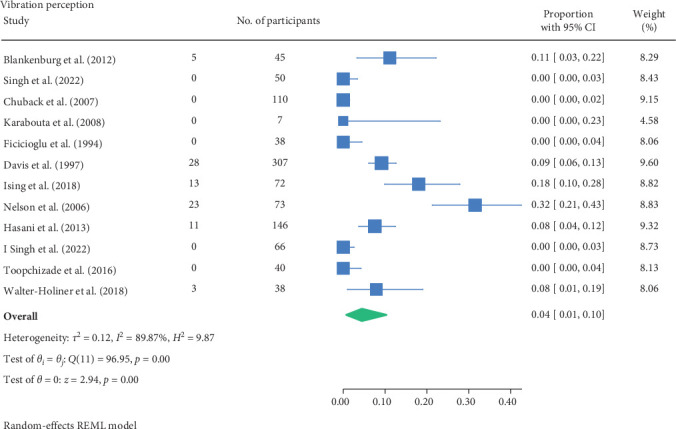
Meta-analysis of abnormal vibration perception events.

The result of the proportion meta-analysis of the 12 studies evaluating abnormal vibration perception events can be seen in Figure 4. The overall (mean) proportion of participants exhibiting an abnormal vibration perception test result was 0.04 with a CI of [0.01, 0.10], indicating that 4% of children with type 1 or 2 diabetes mellitus could exhibit an abnormal vibration perception.

#### 3.1.3. Pain Perception Test

Another assessment of detecting neuropathy is via pain perception using neurotips or temperature.

Studies conducted by Riihima et al. [27], Singh et al. [29] and Toopchizadeh et al. [31] detected no abnormalities in their respective cohorts. This was in contrast to Blankenburg et al. [11] where 45 participants (with mean age of 13.2 years, mean type 1 diabetes duration of 6.7 years and mean HbA1C of 8.28%) had lower pain thresholds to blunt pressure and higher pain thresholds for pinprick pain sensibility compared to the 45 age and gender matched control group. Furthermore, an increased sensitivity to pain occurred upon blunt pressure in 40% of patients and in 20% to pinprick stimuli. This is supported by Karabouta et al. [26] and Fiçicioğlu et al. [23], noting 57.1% and 2.6% having abnormal pain perception, respectively, having a total cohort of 45 participants with a median duration of type 2 diabetes of 1.8 years or mean of type 1 diabetes duration of 3.1 years.

Hasani et al. [24] had also measured pain perception via pin-prick and temperature, which noted 10.3% and 1.4% among a cohort of 146 children (with type 1 diabetes with mean age 11.9 years and mean duration of 3.8 years) had exhibited abnormal results, respectively. Lastly, Blankenburg et al. [11] assessed pain perception via cold and heat pain with a computer-controlled thermotester, where 45 participants (with mean age of 13.2 years, mean type 1 diabetes duration of 6.7 years and mean HbA1C of 8.28%) displayed lower pain thresholds to heat compared to the 45 age and gender matched control group. Moreover, an increased sensitivity to pain occurred upon heat in 13% of patients and in 7% to cold.

None of the studies recorded the participant characteristics of participants nor reported whether any of the characteristics influence abnormal pain perception, except Blankenburg et al. [11], noting a correlation of higher HbA1c with a lower mechanical threshold.

**Figure 5:**
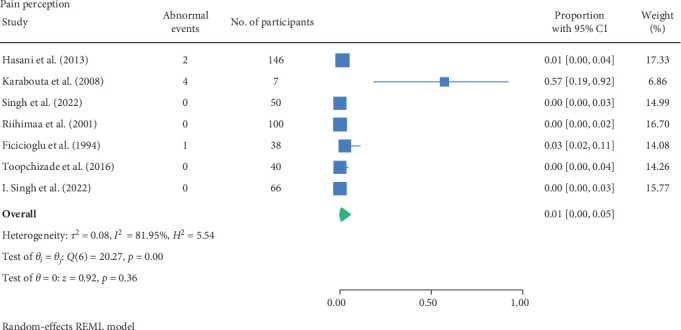
Meta-analysis of abnormal pain perception events.

The result of the proportion meta-analysis of the seven studies evaluating abnormal pain perception events can be seen in Figure 5. The overall (mean) proportion of participants exhibiting an abnormal pain perception test result was 0.01 with a CI of [0.00, 0.05], indicating that 1% of children with type 1 or 2 diabetes mellitus could exhibit an abnormal pain perception.

#### 3.1.4. Thermal Perception Test

Blankenburg et al. [11] noted 24% of 45 participants (with mean age of 13.2 years, mean type 1 diabetes duration of 6.7 years and mean HbA1C of 8.28%) had thermal hypoesthesia; however, this was not statistically significant in comparison to the 45 age and gender matched control group. Moreover, Chuback et al. [19] assessed thermal perception at 25°C and the other at 40°C and found no abnormalities in their participant cohort of 110 participants (with mean age 15 years, mean type 2 diabetes duration 30 months and mean HbA1C of 8.0%). Singh et al. [29], Singh et al. [30] and Toopchizadeh et al. [31] did not specify how the assessment was carried out, however, no abnormalities were recorded.

None of the studies recorded the participant characteristics of participants nor reported whether any of the characteristics influence abnormal thermal perception, except Blankenburg et al. [11], reporting a correlation of higher HbA1c with a higher cold detection threshold.

**Figure 6:**
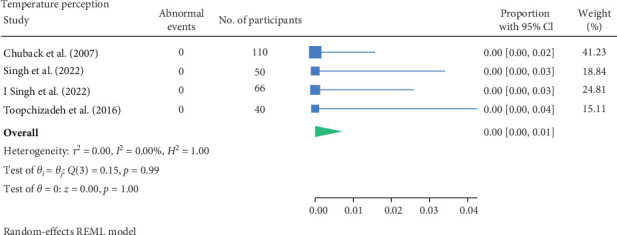
Meta-analysis of abnormal temperature perception events.

The result of the proportion meta-analysis of the four studies evaluating abnormal temperature perception events can be seen in Figure 6. The overall (mean) proportion of participants exhibiting an abnormal temperature perception test result was 0.00 with a CI of [0.00, 0.01], indicating that, based on available evidence, children with diabetes are not expected to exhibit abnormal thermal perception.

#### 3.1.5. Muscle Strength Test and Tone

Muscle testing was performed in five studies [23, 24, 29–31].

Three out of five studies, including Singh et al. [29], Singh et al. [30] and Toopchizadeh et al. [31] noted no abnormalities when performing the testing. This was in contrast to Hasani et al. [24] who reported 23.3% among a cohort of 146 children (with type 1 diabetes with mean age 11.9 years and mean duration of 3.8 years) who displayed muscle weakness. This was supported by Fiçicioğlu et al. [23] noted 7.9% of a cohort of 38 children (with mean age 10.5 years, mean HbA1C of 14.4% and mean type 1 diabetes duration of 3.1 years). It is noted, only Singh et al. [29] had mentioned testing the flexion and extension muscles at the ankle and knee while the other four studies did not specify which muscles were tested. Both Singh et al. [29] and Singh et al. [30] graded muscle strength on a scale of 0–5, where a score of 4 or 5 was considered to be normal, while grade 3 was reduced and grades 0–2 to be severely reduced.

No studies explored muscle tone in this review.

None of the studies recorded the participant characteristics of participants, nor reported whether any of the characteristics were associated with muscle weakness.

**Figure 7:**
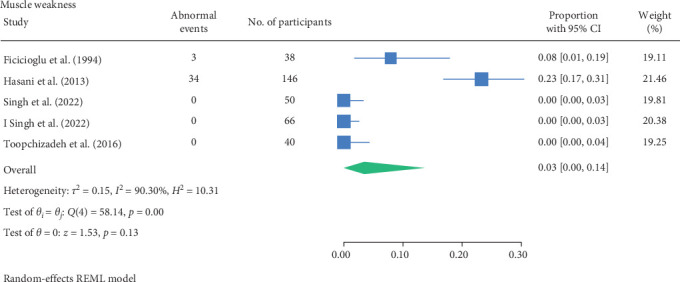
Meta-analysis of muscle weakness events.

The result of the proportion meta-analysis of the five studies evaluating muscle weakness events can be seen in Figure 7. The overall (mean) proportion of participants exhibiting muscle weakness was 0.03 with a CI of [0.00, 0.14], indicating that 3% of children with type 1 or 2 diabetes mellitus could exhibit muscle weakness.

#### 3.1.6. Deep Tendon Reflex

Deep tendon reflexes were tested in eight studies [23, 24, 26, 27, 29–32]. The Achilles (or ankle), quadriceps, gastrocnemius, and biceps and/or triceps surae tendons were assessed. Studies conducted by Karabouta et al. [26] and Singh et al. [29] tested the Achilles tendon and recorded no abnormalities in their respective cohorts. This was in contrast with Walter-Höliner et al. [32], where 5.3% of patients had abnormal ankle reflexes among the 28 participants (with mean age of 12.6 years, mean HbA1C of 8.1% and mean duration of 5.6 years of type 1 diabetes). Riihimaa et al. [27] found 2% of participants with diminished or absent quadriceps, gastrocnemius, and triceps surae tendons reflexes among the 100 participants (with mean age of 13.7 years and mean GHB of 8.5% of type 1 diabetes). This was supported by Fiçicioğlu et al. [23] who noted 18.4% of a cohort of 38 children (with mean age 10.5 years, mean HbA1C of 14.4% and mean type 1 diabetes duration of 3.1 years).

Toopchizadeh et al. [31] had also observed that 47.5% of the 40 participants (with type 1 diabetes of mean age 13 years, mean HbA1C of 7.98% and mean duration of 6.4 years) had reduced deep tendon reflexes in the lower limb. Furthermore, Hasani et al. [24] noted 7% of a cohort of 146 children (with type 1 diabetes with mean age of 11.9 years and mean duration of 3.8 years). Similar to Singh et al. [30], the studies did not specify which tendons were assessed. None of the studies recorded whether participant characteristics were associated with higher rates of abnormal touch perception.

**Figure 8:**
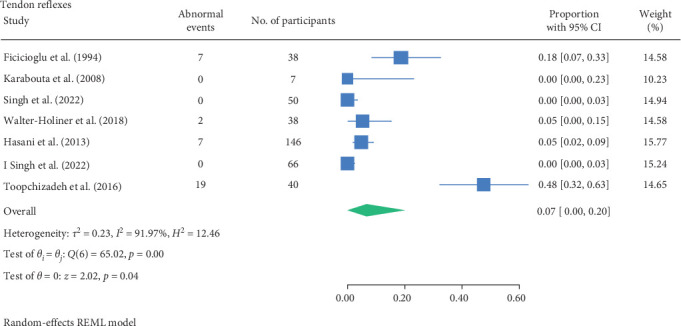
Meta-analysis of abnormal tendon reflexes events.

The result of the proportion meta-analysis of the seven studies evaluating abnormal tendon reflexes events can be seen in Figure 8. The overall (mean) proportion of participants exhibiting abnormal tendon reflexes result was 0.07 with a CI of [0.00, 0.20], indicating that 7% of children with type 1 or 2 diabetes mellitus could exhibit abnormal tendon reflexes.

#### 3.1.7. Joint Position Perception

No abnormalities were recorded in the three studies conducted by Riihimaa et al. [27], Toopchizadeh et al. [31] and Singh et al. [30] in assessing perception of joint position. Only Riihimaa et al. [27] mentioned assessing the first big toe, and the remaining studies did not specify which joints were tested. No studies recorded whether participant characteristics were associated with higher rates of abnormal joint position perception testing.

**Figure 9:**
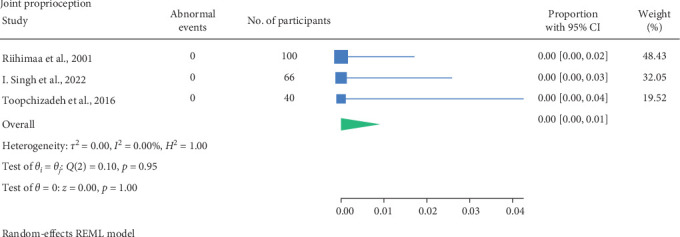
Meta-analysis of abnormal joint proprioception events.

The result of the proportion meta-analysis of the three studies evaluating abnormal joint proprioception events can be seen in Figure 9. The overall (mean) proportion of participants exhibiting an abnormal joint position perception test result was 0.00 with a CI of [0.00, 0.01], indicating that children with diabetes are not expected to exhibit abnormal joint position perception.

#### 3.1.8. Relationship With Variables

Only Chuback et al. [19] studied participants with type 2 diabetes and reported no correlation between duration of diabetes diagnosis, HbA1c, weight or age with the presence or lack of foot abnormalities. Toopchizadeh et al. [31], Walter-Höliner et al. [32], Nelson et al. [18], Ising et al. [25] and Blankenburg et al. [11] reported having no correlation with duration of type 1 diabetes, which is contradicted by Fiçicioğlu et al. [23] and Hasani et al. [24], noting longer disease duration in participants with diabetic neuropathy.

Hasani et al. [24] and Riihimaa et al. [27] observed a higher HbA1c in participants with type 1 diabetes with diabetic neuropathy. This was contradicted by Fiçicioğlu et al. [23], Toopchizadeh et al. [31], Walter-Höliner et al. [32], Cho et al. [20], Davis et al. [21], Nelson et al. [18] and Ising et al. [25]. Walter-Höliner et al. [32] noted no correlation between age, BMI, and gender in participants with type 1 diabetes with diabetic neuropathy. This is supported by Toopchizadeh et al. [31] reporting no correlations in age, Cho et al. [20] reporting no correlations in BMI and Nelson et al. [18] and Ising et al. [25] reporting no correlation in gender in participants with type 1 diabetes with diabetic neuropathy.

### 3.2. Vasculopathy

Overall, four studies described assessments to detect peripheral arterial disease in children with diabetes [5, 6, 19, 26]. Only three clinical assessments were used, which included palpation of pedal pulses, measurement of the ABI and examination for signs and symptoms of peripheral arterial disease.

#### 3.2.1. Pulse Palpation

Among the cohort in Karabouta et al. [26] consisting of two Asian females, four females and one African male (with a median type 2 diabetes duration of 1.8 years and a median age of 14.3 years old), pedal pulses were palpable; however, weak in 57.1% of participants. Chuback et al. [19] also performed this assessment; however, did not provide results in their full text publication and were unable to provide results when contacted.

#### 3.2.2. ABI

Akinyosoye et al. [6] conducted the ABI assessment on a cohort of 45 children with type 1 diabetes with 45 aged and gender matched controls in Southwest Nigeria and found a statistically significant (*p* < 0.001) prevalence of peripheral arterial disease (37.8% vs. 6.7%). The authors had also noted children with type 1 diabetes and peripheral arterial disease had a higher age (mean 11.82 vs., 10.07 years) and HbA1C (mean 13.47% vs. 8.16%) while a negative correlation was found with age, weight, waist circumference, hip circumference, waist to hip ratio and HbA1C with the ABI scores.

This is in contrast with Yu et al. [5] reporting no statistically significant difference (*p*=0.351) in index scores between children with diabetes and matched controls. Yu et al. [5] conducted the ABI assessment on a cohort of 87 children with type 1 diabetes, with 21 sex, age, weight, height and blood pressure matched controls in Taiwan. ABI was classified as low (<0.9), normal (0.9–1.3) and high (>1.3) in Akinyosoye et al. [6], while Yu et al. [5] did not report classifications in ABI.

Currently, there are no studies that have explored the use of a toe–brachial index or absolute toe pressure assessments in children and/or adolescents with diabetes.

#### 3.2.3. Clinical Examination

One study performed a dermatological examination as part of a vascular assessment. These included skin colour and temperature, skin type and texture, signs of infection, oedema, nail problems, foot pulse palpation, and presence of calluses [26]. No significant abnormalities were recorded in Karabouta et al. [26], consisting of 2 Asian females, 4 females and 1 African male (with a median type 2 diabetes duration of 1.8 years and a median age of 14.3 years), apart from 85.7% of participants had displayed areas of callous formation.

Overall, the studies included in this systematic review used a range of clinical assessments and diagnostic criteria when attempting to detect lower limb neurovascular abnormalities in children with juvenile diabetes. This was also the case for study designs and sample sizes used, with some studies not reporting whether their sample was powered.

## 4. Discussion

Among the 18 included studies, 14 explored the prevalence of neuropathy, two investigated neuropathy and vasculopathy combined, and finally, two studies only explored vasculopathy of the lower limb alone. These studies used chair-side clinical assessments to diagnose pathology, such as monofilament, vibration perception, sharp-blunt, temperature perception, reflexes, muscle strength and tone, joint perception, pedal pulses, and ABI.

### 4.1. Neuropathy

There is conflicting evidence regarding lower limb sensory and motor neuropathy in children with diabetes, largely due to variations in methodological design and diagnostic assessments. For instance, the touch perception test varies in the pressure used, test sites, and repetition, while the vibration perception test employs different tools like tuning forks, biothesiometers, and vibrosense metres, with variations in frequencies and foot locations. Inadequate method reporting further complicates the issue, as inconsistent diagnostic criteria lead to varied results. For example, Karabouta et al. [26] recorded insensitivity in one or more sites of a 10 g monofilament as positive, whereas Nelson et al. [18] used a different size monofilament (4.17-U) at the distal hallux.

The proportional meta-analysis revealed varying results across different neurological assessment; 3% may show abnormal touch perception (Figure 3), 4% abnormal vibration (Figure 4), 1% abnormal pain perception (Figure 6), 3% muscle weakness (Figure 7) and 7% abnormal tendon reflexes (Figure 8) while abnormal thermal perception and hallux joint position perception are not expected. This may be due to the involvement of different nerve fibres being tested. For example, in the assessment of large fibre neuropathy, 3% of children experiencing abnormal touch perception (Figure 3) and 4% abnormal vibration perception (Figure 4), both of which assess A-beta fibres where conversely 0% of children exhibiting abnormal joint position proprioception (Figure 9) tests A-Alpha fibres [33, 34]. Abnormal pain and thermal perception (Figure 6), which tests small fibre neuropathy (A-Delta and C fibres), were not as common, potentially due to the unique symptom profile of how pain is perceived in youths [33–35]. It was noted, paraesthesia symptoms were more pronounced, and experiences of pain were fewer or less intense, which contradicts existing adult pain assessments as they disproportionately target pain and have fewer items assessing paraesthesia [35]. Finally, motor function assessments such as abnormal tendon reflexes (7%) and muscle weakness (3%), may show variability, as tendon reflex abnormalities typically emerge earlier in the progression of the disease while muscle weakness tends to develop later on [34, 36].

Only one of the 18 studies was a prospective cohort study design with an observation period of 5 years, where authors concluded a prevalence of 13.2% complications, which increased to 34.2%, a 2.6-fold increase [32]. The authors assessed the neuropathy disability score (NDS) using the ankle reflex, vibration, pinprick, and temperature sensations at the great toe, where an NDS score of 3 or more was considered pathological. Following this, neurological symptoms were scored on neuropathy symptom score (NSS); the nature (burning, numbness, or tingling, fatigue, cramping, or aching), location of symptoms (feet, calves or elsewhere), if symptoms displayed during day and/or nighttime with an extra point if symptoms woke the patient up and relief of pain either/and from walking, standing and sitting/lying down. An NSS of 3 or more indicated diabetic peripheral neuropathy (DPN).

The majority of the studies used a cross-sectional design without age and sex-matched controls, which significantly reduces the ability to detect true pathology in a population of interest over a longer observational period [37]. Furthermore, a wide range of variables, including the duration of diabetes, HbA1c levels, age, gender, BMI, height, weight, and pubertal stage, are shown to have an association in participants with diabetic neuropathy of which are reported inconsistently in each study.

### 4.2. Vasculopathy

There is a paucity of evidence assessing the presence of vascular complications such as peripheral arterial disease in children with diabetes mellitus. One reason may be due to a lack of clinical direction based on current guidelines [14]. One study found that only 6% of general practitioners were aware of the evidence-based guidelines for screening of peripheral arterial disease, and only 5% with the diagnostic guidelines. The high prevalence of peripheral arterial disease using the ABI (37.7% vs. 6.7% in controls) in Akinyosoye et al. [6] could arise due to a higher average value of the HbA1c report in their study [38]. This review found no studies have explored the use of toe–brachial indexes to detect peripheral arterial disease in children with diabetes, which is required given the impact of diabetes on microvascular circulation. Current studies on children have only used ABIs, which have been shown to be less sensitive and specific in detecting peripheral arterial disease in adult people with diabetes [39].

### 4.3. Clinical Implications of Results

The reported proportions of abnormal touch perception (3%), vibration perception (4%), pain perception (1%), muscle weakness (3%), and tendon reflexes (7%) underscore the importance of regular neurovascular screening in children with diabetes. The variability in prevalence estimates across studies suggests a need for standardised diagnostic criteria and assessment methods. Specifically, validating normal and abnormal cutoffs for each lower limb neurological and vascular assessment would aid clinicians in diagnosing abnormalities more accurately. This would enhance identification of at-risk patients and implement early interventions, ultimately improving the quality of life and long-term health outcomes. Moreover, given the increasing prevalence of type 2 diabetes in children, these findings advocate for the integration of routine neurovascular assessments into paediatric diabetes management guidelines.

### 4.4. Strengths and Limitations

#### 4.4.1. Strengths

To our knowledge, this is the first review to collate the evidence of lower limb vasculopathy and neuropathy in children with diabetes using clinical assessments such as the touch perception test, vibration perception test, pain perception test, thermal perception test, muscle strength test, muscle tone, deep tendon reflex, joint position perception, palpation of pedal pulses, measurement of the ABI and examination for signs and symptoms of peripheral arterial disease. It includes papers with a broad geographical reach and participants from 3 to 17 years of age. While some evidence of pathology was identified, this review provides evidence that future high-quality research is required to determine if children with diabetes exhibit lower limb neurovascular complications and whether these complications increase over time.

#### 4.4.2. Limitations

Some limitations to this systematic review should be considered. The quality of the papers included in this review presents a significant limitation to the evidence. Variations in assessment methods and reporting have hindered the ability to evaluate the robustness of the methodologies. Additionally, the lack of prospective case–control studies further undermines the strength and reliability of the findings. Furthermore, disease characteristics such as diabetes duration and severity are factors which influence the prevalence of diabetes complications, and were not measured in this review [4].

This review only included assessment tools typically used in a clinical setting. This review did not include gold standard diagnostic tools, such as NCS to diagnose neuropathy or invasive angiography to diagnose peripheral arterial disease [11, 40]. While NCS are more accurate in detecting nerve fibre disruption, they are rarely used in a paediatric setting due to their painful nature. Out of the 18 studies included, only eight studies performed additional NCS. The papers included in this review used a range of vascular and neurological tests commonly used to screen for peripheral arterial disease and neuropathy in adults with diabetes. The efficacy of many of these tests has yet to be established in paediatric populations [41]. Consequently, their clinical application in children cannot be recommended until rigorous validation studies are conducted.

The limitation of the meta-analyses is the variability in each study's methodology, notably regarding how abnormal events were identified and measured across studies, potentially leading to inconsistent results and reporting.

#### 4.4.3. Implications for Future Research

Based on the results of this review, further research is needed to determine the presentation and prevalence of lower limb neurovascular problems in children with diabetes. Future research should focus on using standardised methods of clinical assessments and reporting the methods of assessment in detail to allow comparability and draw more definitive conclusions. Longitudinal case-control study designs, with larger sample sizes, would greatly strengthen the research base. Lastly, future studies should also aim to validate the diagnostic tests in children, as most assessments have only been validated in adults.

## 5. Conclusion

There is growing evidence that children with diabetes can develop neuropathy and peripheral vascular disease before reaching age 18 years. While the prevalence estimates between studies vary, and not all studies detected pathology, recent studies that did observe pathology provide impetus for clinical caution. Considering the plethora of clinical guidelines recommending routine screening for lower limb neuropathy and vasculopathy in adults with diabetes, the potential benefits of screening in children with diabetes should be evaluated. Early detection of lower limb neuropathy and peripheral vascular disease in children with diabetes may allow early intervention, potentially preventing severe lower limb complications from developing during childhood and persisting into adulthood.

The monofilament and neurotip exhibited the most abnormal results, while the tuning fork, thermal perception, muscle tone and joint position perception recorded low prevalences. There is a paucity of data in assessing the lower limb vascular complications, such as peripheral arterial disease. The prevalence of diabetes in children is increasing, particularly with type 2 diabetes and hence, the potential for increased rates of complications such as neurovascular lower limb problems. Higher-quality evidence will empower policymakers and guideline developers to provide clinicians with robust recommendations for the optimal management of lower limb complications in children with diabetes, ultimately improving patient outcomes.

## Figures and Tables

**Table 1:** Table of included studies.

Article	Aims/objectives	Study design and country	Study sample Includes the mean and standard deviation age, percentage of subjects being female, mean and standard deviation HbA1C%, and duration of diabetes in years and population	Outcome measures and diagnostic criteria	Main findings	Limitations/strength/weakness
Akinyosoye et al. [6]	To investigate the prevalence of peripheral arterial disease in children with type 1 diabetes and to link peripheral arterial disease with clinical characteristics in children with type 1 diabete	Cross-sectional study, Nigeria	45 children (10.73 ± 4.1, 48.9% F) with type 1 diabetes from the paediatric endocrinology clinic of Lagos State University Teaching Hospital and 45 control (10.32 ± 4.0, 48.9% F) from the paediatric outpatient clinic, immunisation clinics and children presenting for medical fitness as a pre-requisite for school resumption	Ankle brachial index was classified as low (<0.9), normal (0.9–1.3) and high (>1.3)	A prevalence of 37.8% of peripheral arterial disease (aged 11.82 ± 3.8, 40.9% being female and HbA1C 13.47% ± 3.2%) in participants and a prevalence of 6.7% of peripheral arterial disease in controls. Those with peripheral arterial disease had a higher age and HbA1C	Small population size. Data collection method was well justified and explained

Blankenburg et al. [11]	To outline the somatosensory dysfunction in children with diabetes, its relationship with HbA1c and duration if diabetes and identify the best screening tool for large-fibre dysfunction	Cross-sectional study, Germany	45 participants (13.2 ± 2.8, 48.9% F, 8.3% ± 1.1%, 6.7 ± 2.5) from the diabetes outpatient department (unspecified) and 45 controls (13.2 ± 2.6, 48.9% F, N/A, N/A) from author's previous study (unspecified)	Quantitative sensory testing using the standardised protocol of the German Research Network on Neuropathic Pain with modifications for children: tactile detection thresholds Vibration detection, cold and warm detection thresholds, mechanical pain threshold and dynamic mechanical allodynia. An abnormal value was identified by calculating the *Z*-score based on age- and gender-specific reference values, where a *Z*-score of >1.96 was defined and hyperalgesia and <1.96 as hypoesthesia or hypoalgesia	Compared to control subjects, patients had: • 3x higher tactile detection thresholds. • No differences for vibration, thermal detection, and cold thresholds • Lower pain thresholds to blunt pressure and heat. • Higher values for pinprick pain sensibility. 31% of the patients had no sensory abnormality, 62% with at least two and 42% with at least three. Tactile hypoesthesia was present in 33% of patients. 11% of the patients had abnormal vibration detection. Thermal hypoesthesia occurred in 24% of patients. In 40% of patients, hyperalgesia occurred to blunt pressure, in 20% to pinprick stimuli, in 13% to painful heat and in 7% to cold	Unspecified location of recruitment. Investigated in one location and one investigator. Neurological assessment justified and explained

Cho et al. [20]	Complication rates from 1990 to 2006, putative risk factors for the first 5 years after diagnosis and whether there us a duration threshold in the first 5 years of diagnosis where complications are more likely to be detected	Retrospective study, Australia	819 participants (14.5 [13.1–15.7], 54% F, 8.5% [7.8–9.5], 4.0 [3.3–4.5]) from the diabetes complications assessment service at the Children's Hospital at Westmead	Thermal threshold testing for hot and cold at the left foot Vibration threshold at the left malleolus and left great toe. An abnormal result was defined as a measure of one or more of the tests outside of the 95^th^ percentile derived from a non-diabetic adolescent, adjusted to height and gender	14% had peripheral nerve abnormalities for the 1990–1994 group, 19% in the 1995–1998 group, 28% in the 1999–2002 group and 23% in the 2003–2006 group. No trend in complications rates across 2–5 years after diagnosis for peripheral nerve abnormalities	Unspecified number of investigation and if they had adapted the same methods into the assessments. Large number of participants

Chuback et al. [19]	To identify the prevalence of foot abnormalities in a cohort of Canadian aboriginal children with type 2 diabetes and its associated risk factors	Cross-sectional study, Canada	110 participants (15 ± 3, 66% F, 8.0% ± 2.0%, 2.5 ± 1.67) from the Pediatric Type 2 Diabetes Clinic at the Children's Hospital of the Health Sciences Centre (affiliated with the University of Manitoba) and the rural outreach clinics for remote northern Manitoba communities	Neuropathic symptoms, 10 g monofilament perception, vibration perception, temperature perception and presence of pedal pulses. The abnormal diagnostic criteria for each assessment were unspecified, however, for data analysis, an abnormal foot was referred to as having paronychia, ingrown toenail or neuropathy	12% had neuropathic symptoms and 0% had abnormal monofilament perception, vibration perception and temperature perception. Abnormalities reported in ages 15 ± 3, HbA1C 8.0 ± 2.0, and 2.67 ± 1.67 years duration of diabetes	Single observer. Unspecified what is defined as neuropathic symptoms. Did not provide data for assessment of pedal pulses

Davis et al. [21]	To establish clinically useful reference ranges for vibration perception threshold (VPT) using the biothesiometer in children and adolescents, to evaluate the reliability of the technique to identify subclinical neuropathy in participants with insulin dependent diabetes mellitus (IDDM), and to examine in a large population-based sample of paediatric patients	Cross-sectional study, Australia	307 IDDM participants (13.3 ± 4.6, 53% F, 10.7 ± 3.5, 5.1 ± 4.9) from the diabetic clinic at Princess Margaret Hospital and 232 non-diabetics (12.9 ± 4.2, 54% F, N/A, N/A) from local schools	VPT (unspecified abnormal diagnostic criteria)	VPTs of patients with diabetes was significantly higher than control subjects. Of 28 abnormal VPTs: • Mean ages 13.4 ± 4.2, 71.4% female, HbA1C 11.2 ± 2.1 and 5.1 ± 3.8 years duration of diabetes. • 16 at both medial malleolus and at the great toe. • 6 at the great toe. • 6 at both medial malleolus.	Single observer. Data collecting method well described. Large sample size

Eppens et al. [22]	To analyse the prevalence of diabetes related complications in children with type 2 compared to type 1 diabetes from 1996 to 2005 and to identify variables related with the development of complications in both groups	Retrospective, comparative clinic-based study, Australia	1433 patients with type 1 diabetes (15.7 [13.9–17.0], 53% F, 8.5% [7.8–9.5], 6.8 [4.7–9.6]) and 68 with type 2 diabetes (15.3 [13.6–16.4], 50% F, 7.3% [6.0–8.3], 1.3 [0.6–3.1]) from the diabetes complications assessment service at the Children's Hospital at Westmead	Thermal and vibration threshold. An abnormal result was defined as a <5% derived from a non-diabetic adolescent control group	1 in 5 patients had peripheral nerve abnormalities	Data collecting method well described. Large sample size

Fiçicioğlu et al. [23]	To assess the prevalence of symptomatic and asymptomatic peripheral neuropathy in children with insulin dependent diabetes mellitus and to evaluate the relationship between peripheral neuropathy and several variables, such as diabetes and metabolic control	Cross-sectional study, Istanbul	38 participants 10.5 ± 3.2 N/A, 14.4% ± 4.7%, 3.1 ± 2.8) with diabetes and 31 control (10.2 ± 2.8 N/A, N/A, N/A)	Muscle strength, tendon reflexes, touch pressure, pain and vibratory sensation testing and sense of joint position. The abnormal diagnostic criteria for each neurological assessment were unspecified. It was scored as to neurological symptoms and neurological disability, where an abnormality neurological examination was considered as either asymptomatic, symptomatic or disabling neuropathy with presence of an abnormal electromyography	8 participants with diabetes had abnormal clinical findings, where 7 had diminished or absent tendon reflexes, 3 had muscle weakness and one had diminished pin-prick sensation at the ankles. One control was diagnosed as having symptomatic diabetic neuropathy. The group with asymptomatic and symptomatic diabetic neuropathy has a mean HbA1C of 20.7% ± 4.9% and 6.1 ± 3.4 years duration of diabetes	No mentions of written consent and ethics approval. Small sample size

Hasani et al. [24]	To examine the prevalence and potential risk factors of peripheral neuropathy (PNP) in children with insulin-dependent diabetes	Cross-sectional study, Iran	146 participants (11.9 ± 3.3, 84% F, N/A, 3.8 ± 2.9) from the Endocrinology and Metabolic Research Centre of Isfahan University of Medical Sciences	Muscle weakness, fine touch, pinprick, pain, temperature, vibration, proprioception, and deep tendon reflexes. The abnormal diagnostic criteria for each neurological assessment were unspecified	27.45 has PNP (aged 12.4 ± 3.3 years with HbA1C 12.62% ± 1.8% and 5.7 ± 3.5 years duration of diabetes), 62.5% were asymptomatic and 37.5% had one of the PNP signs or symptoms. 30.8% of subjects had numbness, 7%–10% showed large myelinated nerve fibre dysfunction and 1.4% had impairment in pain or temperature sensation	Unspecified number of researchers obtaining data

Ising et al. [25]	To assess the vibration perception threshold using a VibroSense Metre and light–touch perception obtained with Semmes–Weinstein's monofilaments in children and adolescents with type 1 diabetes to determine whether subjects with impaired sensation reflects underlying sensory diabetic peripheral neuropathy can be recognised in relation to previously collected normative data. The study also aimed to assess the epidemiological and clinical factors related with reduced vibrotactile perception	Cross-sectional study, Sweden	72 participants (12.8 [11.8–15.0], 45.8% F, 7.3% [6.7–7.8], 5.3 [2.9–8.6]) from the paediatric outpatient clinics at Skåne University Hospital and the Hospital of Helsingborg	Vibration perception threshold and light–touch perception. An abnormal vibration perception threshold was defined when at least three of the frequencies had >1.96 *Z*-scores. An abnormal tactile sensitivity was defined as more then 3.61 (0.4 g)	18% had at least one pathological site on the foot for vibration perception threshold, 6% had at least 2, and 4% had at least 3. None had abnormalities in light–touch perception. None had any clinical signs of neuropathy, such as numbness or pain in foot soles, according to their medical records	Data collected by two researchers. Data collection method well explained and justified

Karabouta et al. [26]	To screen for microvascular issues among adolescents with type 2 diabetes mellitus	Cross-sectional study, United Kingdom	7 participants (14.3 [9.8–17.2], 85.7% F, 6.7% [4.6–9.3], 1.9 [0.8–3]) from the paediatric diabetes clinic (unspecified)	Clinical examination included: colour and temperature, skin type and texture, signs of infection, oedema, nail problems, foot pulse palpation, tendo- Achilles reflexes and presence of calluses. Large nerve fibre function was assessed by vibration threshold and light touch/pressure and small fibre neuropathy. The abnormal diagnostic criteria for vibration threshold were unspecified. Insensitivity in one or more sites were defined as abnormal results for the light touch/pressure threshold and neurotip examination	Normal vibration perception, Achilles' tendon reflexes, palpable posterior tibial pulses. 4 had peripheral neuropathy evident by abnormal large and small nerve fibre function. 5 had weak posterior tibial pulses and 6 had plantar calluses present	Only one tester. Assessments were well explained and justified. Small sample size

Nelson et al. [18]	To compare the outcomes of conventional nerve conduction study with non-invasive methods Vibration perception threshold (VPT) and touch perception threshold (TPT) for detection of peripheral neuropathy in children and adolescents with type 1 diabetes	Cross-sectional study, Canada	73 participants (13.7 ± 2.6, 47.9% F, 9.0% ± 1.0%, 8.1 ± 2.6) from the Alberta Children's Hospital Diabetes Clinic	Diabetic neuropathy symptoms questionnaire, neurologic examination, VPT and TPT. An abnormal touch perception threshold was defined as an insensitivity to the 4.17-U filament. An abnormal vibration perception was defined as an amplitude of more than 0.5 µm	36% had an abnormal neurological examination. Diminished vibration sense in 23 patients and abnormal ankle reflexes in 3 patients. Only 4% had occasional neuropathy symptoms	Evaluators were blinded. Assessments were well explained and justified

Riihima et al. [27]	To investigate the effects of puberty on peripheral nerve function in adolescents with type 1 diabetes	Cross-sectional study, Finland	100 with type 1 children diabetes (13.7 ± 2.0, 51% F, 8.5% ± 1.7%, 7.0 ± 3.5) from the paediatric diabetes outpatient clinic at Oulu University Hospital and 100 controls (13.6 ± 2.0, 51%, N/A, N/A) from nearby schools (unspecified)	Vibration, light touch, pain, joint position and tendon reflexes. The examinations altogether were classified as either present or absent; however, it was unclear what specific findings were used to define each classification	3 patients had symptomatic (tingling or loss of deep tendon reflexes) diabetic polyneuropathy. None had any loss of sensation to light touch, pain, or joint position. There was a slight tendency for an increase in vibration perception threshold with puberty	Unspecified if written or verbal consent was obtained

Shore et al. [28]	To determine whether children with diabetes have functional abnormalities of the microcirculation and, if so, whether these modifications can be shown prior to puberty	Cross-sectional study, United Kingdom	50 participants with diabetes (13.1 ± 2.4, 56% F, N/A, N/A [0.08–14]) and 50 control (11.8 [6–16], 58% F, N/A, N/A) recruited from hospital staff or siblings of participants	Vibration perception threshold (unspecified abnormal diagnostic criteria)	No abnormal vibration perception threshold was observed	Unspecified method of recruitment of participants and whether written consent was obtained

Singh et al. [29]	To assess the prevalence of diabetic peripheral neuropathy (DPN) among children with type 1 diabetes and the role of nerve conduction studies in comparison with clinical examination. Also, the various clinical and biochemical markers that increase the risk of DPN was also investigated	Cross-sectional study, India	50 participants (12.2 ± 2.8, 48% F, 9.4% ± 2.1%, 5.1 ± 2.1) from the paediatric endocrine clinic and neurophysiology lab, at a tertiary level teaching hospital in New Delhi	Neuropathy Symptom (DNS) score, ankle reflex, vibration, cutaneous touch sensation, pain, muscle strength and temperature sensation. Insensitivity to the 10 g monofilament was defined as an abnormal result for the cutaneous touch sensation, while muscle strength was considered reduced and severely reduced in grades 3 and 0–2, respectively. The abnormal diagnostic criteria for vibration and pain were unspecified	No participants had clinical evidence of peripheral neuropathy	Small sample size. Assessments were well outlined

Singh et al. [30]	To determine the prevalence of diabetic polyneuropathy in children with type 1 diabetes and identify related risk factors	Cross-sectional study, India	66 participants (11 ± 3.2, 33% F, 8.7% ± 2.24%, 4 ± 1.8) from the paediatric diabetes clinic and neuro-department of paediatrics the paediatric neurology unit in the Department of Paediatrics at a tertiary-care teaching hospital	Interview of neurological symptoms, touch, superficial pain, and temperature, vibration and joint position sense, muscle strength and deep tendon reflexes. An abnormal muscle power was defined as a grade of less than 4. The abnormal diagnostic criteria for touch, superficial pain, temperature, vibration and joint position were unspecified	None of the children were observed to have symptoms, signs or NSS score to suggest neuropathy	Small sample size. Study did not repeat tests to obtain average

Toopchizadeh et al. [31]	To assess the prevalence and the electrophysiological pattern of subclinical diabetic neuropathy in children and adolescents with type 1 diabetes mellitus based on nerve conduction study	Cross-sectional study, Iran	40 participants (12.73 ± 0.43, 62.5% F, N/A, 6.63 ± 0.25) from the paediatrics clinics of Tabriz University of Medical Sciences, Tabriz, Iran	Muscle force, vibration, temperature, pain, proprioception, fine touch perceptions, pinprick and deep tendon reflexes (unspecified abnormal diagnostic criteria)	There were no reported cases of numbness, muscle weakness, abnormalities of fine touch, pinprick, pain, temperature and vibration proprioception. 47.5% had reduced deep tendon reflexes in the lower limb	Small sample size. Did not mention if written consent was obtained. Did not mention if participant's guardian were obtained. Did not have a control group

Walter-Höliner et al. [32]	To investigate the incidence of diabetic peripheral neuropathy (DPN) using nerve conduction studies (NCS) and compare the diagnostic accuracy of clinical neurological examinations to the gold standard NCV. To evaluate the relationship between NCV with clinical and biochemical indicators that may put patients at risk of DPN. To prospectively evaluate the development of DPN throughout a 5-year follow-up period	Prospective cohort study, Austria	38 participants (12.6 ± 2.4, 44.7% F, 8.1% ± 1.2%, 5.6 ± 3.2) from the paediatric diabetes outpatient clinics of two hospitals (unspecified)	The neuropathy disability score (NDS) including examinations of ankle reflex, light touch, vibration, pinprick, and temperature sensation at the great toe. The examinations were defined as present ( = 0) or absent/reduced ( = 1) for each side. Reflexes were defined as normal ( = 0), present with reinforcement ( = 1), or absent ( = 2). An NDS score of 3 or more was considered pathological. An insensitivity of the 10 g monofilament was considered abnormal	Only 5 patients presented with signs of DPN. 3 patients had loss of pressure sensation, an impairment of vibration perception over the medial malleolus of the ankle bilaterally, and an impaired light–touch perception bilaterally. 2 patients had abnormal ankle reflexes. 4 patients had symptoms of DPN with cramping in calves and feet being the most common sign 13 patients showed signs and symptoms of DPN after 5 years with loss of pressure sensation being the most common sign, followed by impaired vibration perception and abnormal pin-prick testing. Again, cramping in calves and feet being the most common sign	Small sample size. Unspecified population group. Methods well outlined

Yu et al. [5]	To investigate arterial stiffness and establish if an increased brachial–ankle pulse wave velocity might precede macrovascular or microvascular damage	Cross-sectional study, Taiwan	87 participants (13 [10–18], 41.3% F, 9.1% [5.5–13.9], 5.125 [0.708–13]) from the Paediatric Outpatient Departments of Chang Gung Children's Hospital, Link-Kou Medical Center, Taiwan and 21 control participants (12 [11–15], 38% F, N/A, N/A)	Ankle brachial index (abi) and brachial ankle pulse wave velocity (baPWV). ABI had unspecified abnormal diagnostic criteria	No statistical difference between ABI and baPWV in both groups. Suggests that arterial stiffness does not increase in the early stages of uncomplicated type 1 diabetes in the teens	Did not mention if written consent was obtained. Did not mention if participant's guardian were obtained. Unspecified where control subjects were recruited. Did not provide number of patients experiencing abnormal ABI and baPWV

## Data Availability

The data that support the findings of this study are available from the corresponding author upon reasonable request.

## Supporting Information

Supporting Information 1 Table S1. Ovid SP Medline search strategy.

Supporting Information 2 Table S2. Table of excluded studies.
