# Supplementary material for: Prevalence and Presentation of Lower Limb Neurovascular Complications in Children With Diabetes: A Systematic Review With Proportion Meta-Analysis
Source: Pediatr Diabetes. 2025 Jun 23;2025:7664860. doi: 10.1155/pedi/7664860 (PMC12208751; doi:10.1155/pedi/7664860)
Supplement: Supporting Information 1 — Table S1. Ovid SP Medline search strategy. [file 7664860.f1.docx]

Supplementary Table 1. Ovid SP Medline search strategy

| 1 | Diabetes Mellitus, Type 2/ or Diabetes Mellitus, Type 1/ or diabet*.mp. or Diabetes Mellitus/ |
| --- | --- |
| 2 | juvenile diabet*.mp. |
| 3 | Child, Preschool/ or Child/ or child*.mp. |
| 4 | Adolescent/ or adolescent.mp. |
| 5 | lower limb.mp. or Lower Extremity/ |
| 6 | ankle.mp. or Ankle/ |
| 7 | Foot/ or Diabetic Foot/ or foot*.mp. |
| 8 | feet.mp. or Foot/ |
| 9 | neuropath*.mp. or Diabetic Neuropathies/ |
| 10 | Peripheral Vascular Diseases/ or Peripheral Arterial Disease/ or vasculopath*.mp. |
| 11 | diabetic foot ulcer.mp. |
| 12 | 1 OR 2 |
| 13 | 3 OR 4 |
| 14 | 5 OR 6 OR 7 OR 8 OR 9 OR 10 OR 11 |
| 15 | 12 AND 13 AND 14 |
| 16 | limit 15 to "all child (0 to 18 years)" |
