# Supplementary material for: Prevalence and Presentation of Lower Limb Neurovascular Complications in Children With Diabetes: A Systematic Review With Proportion Meta-Analysis
Source: Pediatr Diabetes. 2025 Jun 23;2025:7664860. doi: 10.1155/pedi/7664860 (PMC12208751; doi:10.1155/pedi/7664860)
Supplement: Supporting Information 2 — Table S2. Table of excluded studies. [file 7664860.f2.docx]

Supplementary Table 2. Table of Excluded Studies

| **Reasons of exclusion** | **Wrong outcome** | **Wrong study design** | **Conference abstract only** | **Wrong patient population** | **Unable to retrieve full text or contact author.** |
| --- | --- | --- | --- | --- | --- |
| Description | Dermatological and musculoskeletal related complications. Studies that used sub-clinical examination such as Nerve Conduction Studies. | Ineligible study designs such as Case control studies and conference abstracts. | Poster Abstract only of a conference. No full text available. | Adults aged 18 and above. Any additional endocrinological, rheumatological or other medical conditions that may impact on lower limb neurovascular status. | Poster Abstract. |
| Citations | Abbas et al. (2016), Abd El Dayem et al. (2015), Abuelwafaa et al. (2019), Agius et al. (2023), Ahmadov (2014), Al-Kufi et al. (2020), Al-Mutairi et al. (2010), Banerjee et al. (2023), Barkai et al. (1998), Barnett et al. (1995), Belch et al. (1996), Blankenburg et al. (2012), Bogusz-Gorna et al. (2023), Boopathy et al. (2023), Bradley et al. (2016), Cenesiz et al. (2003), Chiumello et al. (1989), Chiumello et al. (1989), Craig et al. (2008), Dahlquist et al. (2008), DiMeglio (2011), Dorchy & Loeb (1986), Dorchy et al. (1985), Duffin et al. (1999), Duffin et al. (1999), Duffin et al. (2003), Duffin et al. (2003), Duffin et al. (2002), Duffin et al. (2002), Edidin (1985), Ficicioglu et al. (1994), Fiçicioğlu et al. (1996), Francia et al. (2018), Francia et al. (2016), Francia et al. (2013), Frohlich-Reiterer & Borkenstein (2010), Gallai et al. (1988), Heimans et al. (1987), Jangir et al. (2023), Karachaliou et al. (1996), Karahanyan et al. (1994), Karavanaki & Baum (1999), Katsalouli et al. (2010), Kaya Mutlu et al. (2018), Khan et al. (2000), Kirmizibekmez et al. (2014), Lawnicki et al. (2019), Lee et al. (2003), Lotosh et al. (2012), Louraki et al. (2016), Ludvigsson et al. (1979), Meh & Denislic (1980), Meli et al. (1989), Moser et al. (2013), Msanga et al. (2020), Msanga et al. (2020), Natriashvili & Chkhartishvili (2010), Pacaud et al. (2022), Rasmussen et al. (2023), Romano et al. (1998), Rosenbloom (1990), Rosenbloom et al. (1981), Sandhu et al. (2020), Schickenberg-Werrij et al. (2014), Seddon & Smith (1988), Seth et al. (2017), Sochett & Daneman (1999), Solders et al. (1997), Solntsava & Zagrebaeva (2013), Stefaniak et al. (2020), Tan et al. (1985), Toopchizadeh et al. (2016), Torigoe et al. (1999), Turgut et al. (2004), Virk et al. (2016), Vukovic et al. (1996), Vuković et al. (1996) and Zhang et al. (2022). | Ahel et al. (2019), Albasri & Al-Sofiani (2022), Amutha & Mohan (2016), Ayanoglu et al. (2022), Bertalan & Gregory (2011), Bhansali et al. (2004), Chiumello et al. (1984), Chrzanowska et al. (2023), Claus et al. (1993), Daneman (2005), Dayal et al. (2016), Donaghue et al. (2009), Donaghue et al. (2014), Elbarbary et al. (2014), Ficicioglu et al. (1996), Fröhlich-Reiterer & Borkenstein (2010), Giza et al. (2020), Giza et al. (2020), Glastras et al. (2005), Globa & Zelinska (2010), Graves & Donaghue (2020), Graves & Donaghue (2020), Greenwood & Traisman (1971), Gunaid (2018), Hamilton et al. (2004), Hamilton et al. (2004), Hannon et al. (2005), Holiner et al. (2013), Jafari et al. (2022), Jaeger et al. (2022), Jin et al. (2023), Kallinikiou et al. (2019), Kallinikiou et al. (2019), Kumar & Ghatak (2010), Louraki et al. (2012), Marcovecchio & Chiarelli (2011), Marcus et al. (1973), Margolis et al. (1969), Moser et al. (2011), Novikova et al. (2012), Özgüç Çömlek et al. (2022), Pena et al. (2020), Rangel et al. (2015), Rasmussen et al. (2021), Rasmussen et al. (2021), Rasmussen et al. (2021), Rubio Cabezas & Argente Oliver (2007), Samahy et al, (2015), Shafi & Latief (2017), Shafi & Latief (2017), Shareef et al. (2017), Siok-Hoon et al. (1985), Sochett & Daneman (1999), Sørensen & Aagenaes (1988), Sun et al. (2020), Tan et al. (1985), Trotta et al. (2004), Trotta et al. (2004), Vasigh et al. (2021) and Vinik (2006). | Abuelwafaa et al. (2017), Ahmed et al. (2017), Ahmed (2018), Ahmed et al. (2017), Delonga et al. (2012), Francia et al. (2020), Globa & Zelinska (2014), Globa & Zelinska (2011), Hassan et al. (2012), Ising et al. (2017), Jaiswal et al. (2014), Kumar et al. (2021), Oberhauser et al. (2022), Rasmussen et al. (2022), Robinson et al. (2017), Salem et al. (2010), Tinti et al. (2021) and Walter-Holiner et al. (2017). | Amutha et al. (2017), Amutha et al. (2021), Bao et al. (1999), Bertora et al. (1998), Bideci et al. (2006), Binns-Hall et al. (2019), Blankenburg et al. (2010), Broser et al. (2023), Cabezas-Cerrato (1998), Campea et al. (1989), Christensen et al. (2017), Costantini et al. (2014), Coutinho Dos Santos et al. (2002), Donaghue et al. (1996), Donaghue et al. (1993), Dyck et al. (2011), Ferreira et al., (2005), Fischer et al. (1979), Ghaemi et al. (2018), Giani et al. (2019), Gomes et al. (2021), Gomes et al. (2021), Hajas et al. (2017), Hajas et al. (2016), Hoeliner et al. (2012), Hyllienmark et al. (2013), Hyllienmark et al. (2013), Hyllienmark et al. (1995), Jaiswal et al. (2017), Jaiswal et al. (2013), Kamaleldeen et al. (2018), Kapellen et al. (2003), Karsidag et al. (2005), Khan et al. (2000), Lee et al. (2010), Lièvre et al. (2005), Ludvigsson et al. (2011), Mayr et al. (1986), Moser et al. (2012), Mrad et al. (2000), Najem et al. (2021), Najem et al. (2021), Nordwall et al. (2006), Oberhauser et al. (2022), Oberhauser et al. (2023), Oberhauser et al. (2023), Olsen et al. (1994), Oyenusi et al. (2014), Parada et al. (2013), Shah et al. (2018), Shalitin et al. (2002), Sophausvaporn et al. (2023), Sophausvaporn et al. (2023), Stafford et al. (2019), Sukumar et al. (2022), Turkyilmaz et al. (2017), Van der Heyden et al. (2013), Weintrob et al. (2007) and Zander et al. (2002) | Almenabbawy et al. (2019), Arici et al. (2015), Bognetti et al. (1997), Briceno et al. (2011), Campea et al. (1991), Comi et al. (1986), Comi et al. (1986), Demirel et al. (2013), Hajas & Jakubicka (2010), Hernandez Cossi et al. (1976), Kaar et al. (1983), Karpovich et al. (1999), Kazibutowska (1974), Louraki et al. (2009), Maccarone et al. (2002), Madjova et al. (2003), Mayr et al. (1986), Moglia et al. (1994), Moglia et al. (1994), Radtke (1960) and Tshukova Bojinova et al. (1974). |
